# Are GABAergic drugs beneficial in providing neuroprotection after traumatic brain injuries? A comprehensive literature review of preclinical studies

**DOI:** 10.3389/fneur.2023.1109406

**Published:** 2023-02-02

**Authors:** Shyam Kumar Sudhakar

**Affiliations:** Division of Sciences, School of Interwoven Arts and Sciences, Krea University, Sri City, Andhra Pradesh, India

**Keywords:** GABA, traumatic brain injuries (TBI), propofol, isoflurane, neuroprotection

## Abstract

Traumatic brain injuries (TBI) caused by physical impact to the brain can adversely impact the welfare and well-being of the affected individuals. One of the leading causes of mortality and dysfunction in the world, TBI is a major public health problem facing the human community. Drugs that target GABAergic neurotransmission are commonly used for sedation in clinical TBI yet their potential to cause neuroprotection is unclear. In this paper, I have performed a rigorous literature review of the neuroprotective effects of drugs that increase GABAergic currents based on the results reported in preclinical literature. The drugs covered in this review include the following: propofol, benzodiazepines, barbiturates, isoflurane, and other drugs that are agonists of GABA_A_ receptors. A careful review of numerous preclinical studies reveals that these drugs fail to produce any neuroprotection after a primary impact to the brain. In numerous circumstances, they could be detrimental to neuroprotection by increasing the size of the contusional brain tissue and by severely interfering with behavioral and functional recovery. Therefore, anesthetic agents that work by enhancing the effect of neurotransmitter GABA should be administered with caution of TBI patients until a clear and concrete picture of their neuroprotective efficacy emerges in the clinical literature.

## Introduction

Traumatic brain injuries (TBI) are a major public health problem both in India (1) and the United States (2). Physical injury to the brain in numerous forms can cause TBI and this may lead to the death of neurons and other cells in the affected region ultimately resulting in loss of function (3). TBI can potentially lead to the development of long-term neurological, and psychiatric problems in the affected individuals (4–6). Therefore, TBI and associated co-morbidities could severely disrupt the quality of life of the affected individuals hindering their ability to function independently (5).

Being one of the major causes of death and dysfunction in the United States, the number of individuals living with TBI-related ailments is 5.3 million and it is estimated that the number of people who die from TBI-related complications is around 50,000 annually in the United States (2). Due to the medical complications that one could face post head injury, TBI could potentially stress the healthcare systems and impose a hefty financial burden. The average cost of treating individuals affected by TBI is estimated to be around $50 billion annually in the United States (2). In India, the incidence of TBI is 1.6 million annually based on epidemiological data (1). Additionally, death due to head injury accounts for 200,000/year, and about 1 million will need access to rehabilitation services (1). Therefore, TBI and associated complications create a huge socioeconomic burden.

Neuronal damage after TBI can be attributed to primary and secondary injuries each employing a distinct set of pathophysiological mechanisms (7, 8). Primary injury is due to the death of neurons, non-neurons, and blood vessels at the site of physical impact leading to energy deficiency (7). On the other hand, secondary injury could happen over days, months, or even years after a primary traumatic impact. Secondary brain injury is due to a complex set of signaling cascades and mechanisms that ultimately result in membrane depolarization, excitotoxicity, and activation of pathways leading to programmed cell death (7). While the loss of tissue due to primary brain injury is generally irreversible, secondary brain injuries can be prevented by administering the right therapeutic interventions immediately after the primary injury. Termed “golden hours,” the first (9, 10) few hours post TBI when the post-traumatic excitotoxicity reaches the peak, is crucial for causing neuroprotection, reducing secondary brain injuries, and aiding long-term functional recovery. Therefore, therapeutic interventions for TBI might need to target this crucial time frame in order to achieve maximal efficacy. Unfortunately, numerous clinical trials in quest for an effective neuroprotective agent in TBI have failed and there is no cure (11) till date which can be partly attributed to the heterogeneity of injury types in TBI (12, 13). However, robust clinical care and patient management post TBI could reduce the damage inflicted by secondary brain injuries and offer valuable neuroprotection to the affected individuals.

TBI patients need to go through anesthesia for various reasons such as prevention of seizures, pharmacological sedation, and surgery (14, 15). In clinical TBI, sedation through drugs still remains the first line of treatment to prevent further complications, normalize intracranial pressure (ICP), and reduce metabolic demand (14, 16). Generally, the choice of anesthetic agents is decided by the treating physician based on the drug's hemodynamic factors, its ability to reduce ICP, cerebral metabolic rate, and the drug's potential to cause short-term and long-term side effects (15, 17, 18). Unfortunately, one factor that is often under-emphasized while selecting an anesthetic agent in clinical TBI is the ability of the drug to prevent cell death and reduce histological damage. This could be due to the lack of drug efficacy data in the clinical literature and ethical concerns about experimentation on humans. There are several pre-clinical animal research studies that state that the choice of anesthetic agents could affect the extent of secondary injuries post TBI (19–23). Such animal studies could come to the rescue and offer valuable data on the ability of various drugs used as anesthetic agents in clinical TBI to cause neuroprotection.

**Figure 1:**
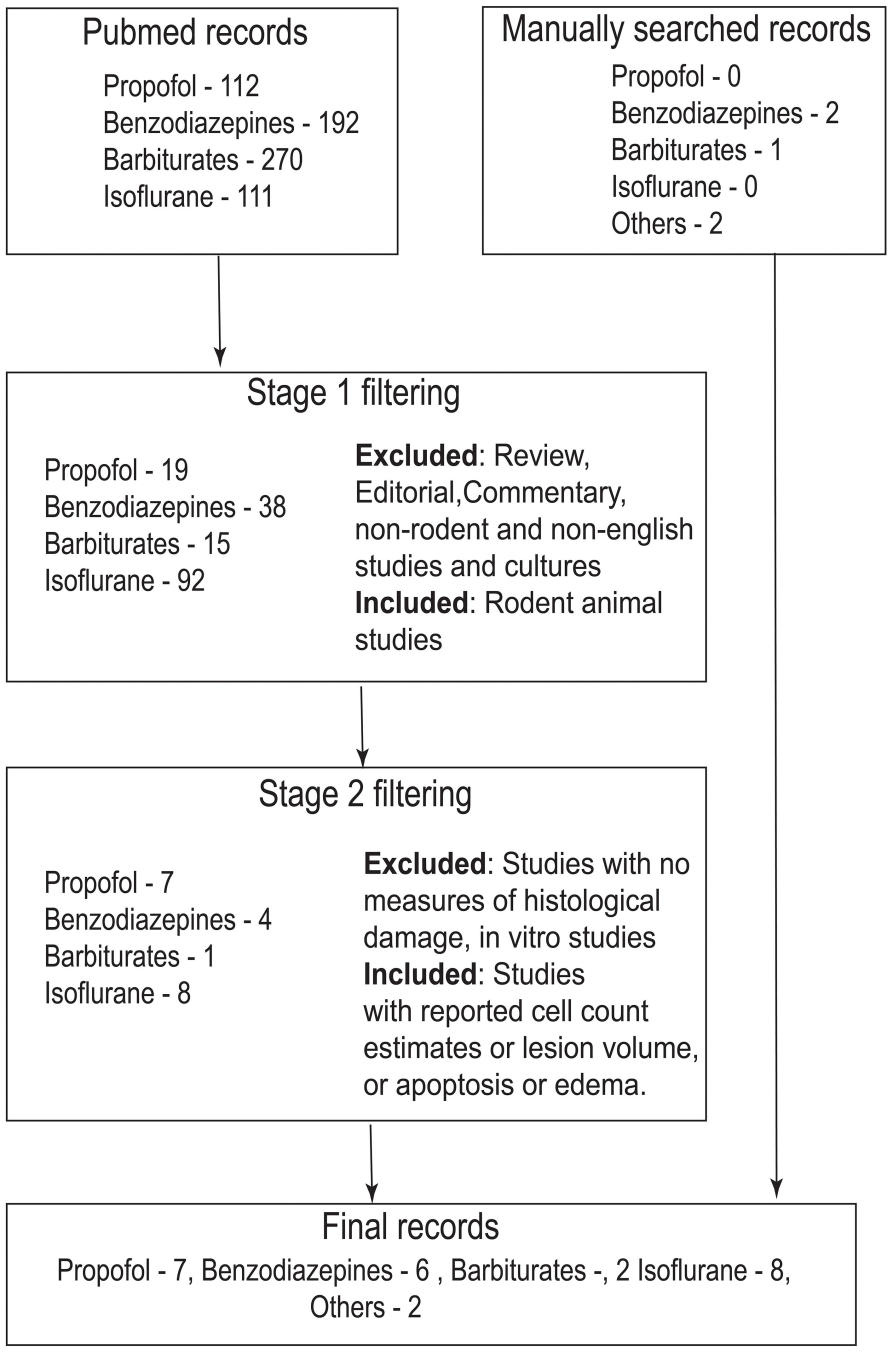
Flowchart that describes search strategy and inclusion/exclusion criteria for the study. Records were searched in PubMed (https://pubmed.ncbi.nlm.nih.gov/) using the search term “traumatic brain injury” + drug name (For example, the search term for propofol would be traumatic brain injury propofol). In stage 1, abstract of the records were screened. Only rodent animal studies were included in this stage. Non-rodent studies, reviews, commentaries, editorials and non-English articles were excluded. In stage 2, full text of the articles were screened according to the inclusion criteria mentioned in the figure. Records that have passed through stage 2 filtering along with manually cross-referenced records were included in the manuscript.

Here, in this study, I have reviewed the neuroprotective efficacy of a specific class of drugs that augment GABAergic neurotransmission (GABA_A_ receptor agonists) from preclinical animal research studies. GABA_A_R agonists are commonly employed as anesthetic agents in clinical TBI owing to their safety profile and anti-epileptic efficacy (3, 16, 18, 24). After performing an exhaustive literature search, only studies that reported direct metrics on histopathological damage or edema were included in the review (Figure 1). TBI studies that measure the effect of GABAergic drugs on neuroinflammation were excluded from this review because inflammation may not always be neurotoxic and may even be useful especially in the acute stages following TBI (25–29).

Based on the data available in the pre-clinical studies, I find that GABA_A_R agonists not only fail to offer neuroprotection but also can impede functional recovery post TBI. Clinical trials need to be conducted to study the potentially deleterious effects of GABA_A_R agonists, especially in severe TBI cases. Until a clear picture emerges about the neuroprotective properties of GABA_A_R agonists in clinical TBI, one might need to avail caution and consult the efficacy data available in the scientific literature of pre-clinical animal studies.

## Propofol

Propofol is one of the widely used anesthetic agents in clinical TBI owing to its relatively well-documented safety profile, quick time scale of action and well-established neurophysiological mechanisms (14). Propofol exerts its action by augmenting the activity of chloride currents through GABA_A_Rs and also blocks voltage-gated sodium channels (14, 30). However, several preclinical TBI studies (19, 31–34) have highlighted the inefficacy of propofol in causing neuroprotection and promoting functional recovery.

In a study that quantified the neuroprotective efficacy of different drugs commonly used as anesthetics in clinical TBI, the authors report that the application of propofol post controlled cortical impact (CCI) in rats did not have any effect on the lesion volume and the number of remaining CA1 neurons in the hippocampus of the injured brain (19). Also, propofol administration impaired the recovery of motor function measured by beam balance test during the first few days after TBI. Further, propofol did not have any effect on cognitive function outcome measured using the Morris water maze (MWM) test at 14–18 days post injury. In another study, propofol treatment at 24-h post CCI in rats increased the injury size and impaired motor function outcome at 30 days post injury (32). Thal et al. (31) employing the same method (CCI) for inducing TBI have shown that propofol not only had a null effect on the lesion size post TBI but also impaired the extent of functional recovery and reduced neurogenesis. A similar result was also reported in another study (33) where propofol infusion didn't have any effect on lesion volume and eosinophilic cell count in the hippocampus both at low or high doses post CCI in rats.

Even though the above studies have established the potentially detrimental effect of propofol on neuroprotection through animal experiments, there are a few reports in the literature that state that propofol could cause neuroprotection especially when applied prior to TBI. In a study (35) that involved fluid percussion injury (FPI) in rats, propofol treatment prior to TBI significantly reduced the lesion volume and promoted functional recovery. Similar effects of propofol could be seen in a study (36) where the drug was administered soon after (10 min) inflicting TBI to animals through CCI. Additionally, propofol administered at various time points post TBI reduced cell death in the surrounding regions that received primary impact (37).

Therefore, one may be of the opinion that purely from a neuroprotection perspective in reducing lesion size and inducing functional recovery, propofol might not be helpful and could potentially be detrimental especially when applied post TBI in experimental animals (Table 1).

**Table 1:** List of preclinical studies involving drugs that target GABAergic neurotransmission in TBI.

**Drug**	**Injury model and animal groups**	**Drug administration timeline**	**Effect on histological outcome and functional recovery**
Propofol (19)	CCI Sprague-Dawley rats *n* = 9 for each experimental drug group (diazepam, fentanyl, morphine, isoflurane, ketamine, pentobarbital, ketamine, no anesthesia and sham)	Drug administration for 1-h post TBI.	No effect on histological outcome (lesion volume and surviving hippocampal neurons measured at 21 days post TBI). Propofol administration affected motor function recovery (first 5 days post TBI) but had no effect on cognitive recovery (14–20 days post TBI).
Propofol (32)	CCI C57BL/6 mice *n* = 10 for each group (propofol at 6 or 24-h and vehicle or saline)	Single bolus of propofol administration post TBI (6–24-h).	Increased lesion volume observed at 72-h post CCI in the propofol treated cohort. Propofol administration impaired recovery of locomotor function (gait analysis at 30 days post TBI).
Propofol (31)	CCI Sprague-Dawley rats *n* = 10–17 for each group. Groups tested were sham (low and high dose) and CCI (low and high dose)	Drug administration during (for 2-h, 30 min before and 90 min after) or post TBI (for 3-h at 2-h post injury).	Propofol had no effect on the lesion size (28 days post insult). Propofol impaired recovery of neurological function at 28 days post insult in a dose-dependent manner.
Propofol (33)	CCI Sprague-Dawley rats *n* = 9–10 for each group. Groups tested were CCI (propofol high and low dose), CCI (halothane) and sham	Drug administered post TBI for 6-h.	Propofol (at both doses) did not exert any effect on the lesion volume and eosinophilic cell count in the hippocampus (at 6-h post insult).
Propofol (35)	FPI Sprague-Dawley rats *n* = 6–8 for each group. Groups tested were FPI (propofol and isoflurane) and sham	Continuous propofol infusion before TBI induction.	Propofol decreased the lesion volume (at 28 days post TBI induction) compared to isoflurane anesthesia. Propofol aided the recovery of cognitive function in novel object recognition task at 21 days post TBI.
Propofol (36)	CCI Sprague-Dawley rats TBI + drug (*n* = 10) TBI + no drug (*n* = 10) Sham + saline (*n* = 8) No TBI + drug (*n* = 10)	Intra-peritoneal injection given at 10 min post TBI.	Propofol reduced formation of cerebral edema (estimated at 12-h post TBI).
Propofol (37)	CCI Sprague-Dawley rats *n* = 9 for each of the seven experimental groups as mentioned in the paper	Propofol delivered at 1, 2, and 4-h post TBI through intra-peritoneal injection and followed by 2-h infusion.	Propofol reduced cell death in the peri-contusional cortex at 24-h post CCI.
Diazepam (19)	CCI Sprague-Dawley rats *n* = 9 for each experimental drug group (diazepam, fentanyl, morphine, isoflurane, ketamine, pentobarbital, ketamine, no anesthesia and sham)	Drug administration for 1-h post TBI.	No effect on histological outcome by diazepam (lesion volume and surviving hippocampal neurons) at 21 days post TBI. Diazepam administration affected cognitive recovery assessed through MWM test at 14–20 days post TBI.
Diazepam (38)	CCI C57BL/6J mice TBI + drug (*n* = 13) TBI + vehicle (*n* = 13) Sham + drug (*n* = 14) Sham + vehicle (*n* = 12)	Continuous drug infusion for 1-week post TBI through an osmotic pump.	Diazepam did not have any effect on tissue loss or number of degenerating cells at 3 days post injury.
Midazolam (39)	CCI C57BL/6 mice *n* = 9–11 for each group (TBI + saline, TBI + low dose midazolam, TBI + high dose midazolam and TBI + high dose midazolam + flumazenil)	Single point drug administration at 24-h post TBI.	Midazolam did not have any effect on the lesion volume measured at 72-h post TBI. Midazolam impaired neurological recovery assessed through NSS score (40) at 72-h post injury.
Diazepam (22)	Electrolytic lesion. Long Evans hooded rats *n* = 16 for TBI group and *n* =8 for sham group. In each group half of the animals were undrugged.	Drug administration at 10–12-h post TBI and continued till 22 days (intra-peritoneal injection).	Diazepam had no effect on the lesion size. Diazepam impaired recovery from sensory asymmetry as long as 22 days post injury.
Diazepam (41)	Electrolytic lesion. Long Evans hooded rats *n* = 9 and 7 for TBI (anterior-medial neocortex) groups receiving diazepam and vehicle, respectively. *n* = 9 and 9 for TBI (sensorimotor neocortex) groups receiving diazepam and vehicle, respectively. *N* =14 for sham operated animals	Drug administration at 10–12-h post TBI and continued till 21 days (intra-peritoneal injection).	Increased atrophy of the striatum and cell death in the substantia nigra pars reticulata was observed in diazepam treated injured (anterior-medial cortex) animals. Diazepam treatment impaired recovery from sensorimotor asymmetry as long as 91 days post injury following anterior-medial cortical lesion.
Diazepam (42)	Central FPI Sprague-Dawley rats *n* = 8 each for TBI + diazepam, TBI + saline and sham operated controls (pretreatment). For post-treatment, *n* = 6 for TBI + diazepam and *n* = 5 for TBI + saline.	Drug administration at 15 min prior or post TBI induction (intra-peritoneal injection)	Rats that were subjected to diazepam pretreatment had reduced mortality rates. Both pre and post treatment with diazepam assisted in cognitive recovery assessed through MWM test at 10–15 days post TBI.
Pentobarbital (19)	CCI Sprague-Dawley rats *n* = 9 for each experimental drug group (diazepam, fentanyl, morphine, isoflurane, ketamine, pentobarbital, ketamine, no anesthesia and sham)	Drug administration for 1-h post TBI.	No effect on histological outcome after pentobarbital administration at 21 days post TBI. Pentobarbital did not help in recovery of motor or cognitive function assessed at various time points post TBI.
Phenobarbital (23)	Electrolytic lesion. Long Evans hooded rats for three groups: TBI + low dose (*n* = 3), TBI + high dose (*n* = 4) and TBI + NaCl (*n* = 6)	Drug administration (2 times a day) at 48-h for up to 7 days post TBI (intra-peritoneal injection).	Phenobarbital treatment did not have any effect on the lesion volume. Phenobarbital treatment impaired recovery from sensorimotor asymmetry as long as 45 days post injury (no dose-dependency noticed).
Isoflurane (19)	CCI Sprague-Dawley rats *n* = 9 for each experimental drug group (diazepam, fentanyl, morphine, isoflurane, ketamine, pentobarbital, ketamine, no anesthesia and sham)	Drug administration for 1-h post TBI.	Isoflurane administration led to better hippocampal neuronal survival rates at 21 days post TBI. Isoflurane resulted in recovery of cognitive function assessed by MWM test at 14–20 days post TBI.
Isoflurane (21)	CCI Sprague-Dawley rats *n* = 9 for each experimental drug group (isoflurane and fentanyl) and (*n* = 6) for each sham anesthetic group	Continuous drug administration before TBI and continued till 3.5–4-h post TBI.	Isoflurane resulted in better hippocampal neuronal survival rates (estimated at 21 days post TBI) compared to fentanyl although edema and ICP were similar. Isoflurane resulted in superior motor (1–5 days) and cognitive function recovery (14–20 days) compared to fentanyl.
Isoflurane (43)	CCI Sprague-Dawley rats *n* = 9 for each group tested (isoflurane, fentanyl and recovery with no anesthesia)	Continuous drug administration before TBI and continued till 1-h post TBI.	Compared to fentanyl, isoflurane resulted in better hippocampal neuronal survival rates (21 days post injury). Isoflurane resulted in better functional recovery compared to fentanyl (1–20 days post injury).
Isoflurane (20)	CCI C57BL/6N mice *n* = 6 for each group tested [isoflurane, sevoflurane and combo (midazolam, fentanyl, medetomidine)]. Two such cohorts were utilized for histology at 15 min and 24-h post TBI	Continuous anesthesia initiated before TBI and stopped right after injury induction.	Isoflurane resulted in reduced contusional volume measured at 24-h post injury. Isoflurane also resulted in better recovery of neurological function measured by NSS test (40) at 24-h post TBI.
Isoflurane (44)	CCI C57BL/6J mice receiving avertin (*n* = 78) and isoflurane (*n* = 57) anesthesia	Animals received a single impact or 2–3 repeated impacts with 48-h gap. Anesthesia applied prior to surgery.	Isoflurane resulted in reduced axonal injury compared to avertin anesthesia at 24-h post TBI.
Isoflurane (45)	CCI Rats TBI + short anesthesia (*n* = 20) TBI + long anesthesia (*n* = 30)	Short anesthesia for 30 min (at 7.5-h post TBI) and longer anesthesia for 4 (at 4-h post TBI) hours was administered.	Prolonged anesthesia resulted in higher edema formation immediately after injury.
Isoflurane (46)	CCI C57BL/6J mice *n* = 7 for each group mentioned below: CCI + isoflurane CCI + sevoflurane Sham + isoflurane Sham + sevoflurane	Anesthesia was started prior to CCI and continued for 15–20 min during injury induction.	Edema formation was higher in isoflurane treated animals compared to sevoflurane at 24-h post TBI.
Isoflurane (47)	CCI Sprague-Dawley rats CCI + isoflurane (normal sedation, *n* = 12) CCI + isoflurane (deep sedation, *n* = 12)	Anesthesia was maintained for 2-h prior to CCI.	Deep anesthesia resulted in increased neurodegeneration and poor functional performance estimated at 48-h post TBI.
Topiramate (48)	Lateral FPI Sprague-Dawley rats TBI + drug (*n* = 35) TBI + saline (*n* = 25) Sham + drug (*n* = 21) Sham + saline (*n* = 26)	Drug given at 30 min, 8, 20 and 32-h post TBI (intra-peritoneal injection).	Topiramate did not have any effect on the volume of the contusional tissue (1-month post TBI) and edema formation (48-h post TBI). Topiramate resulted in better recovery of motor function (4 weeks post TBI) but affected cognitive learning (4 weeks post TBI).
Vigabatrin (49)	Electrolytic lesion. Long–Evans hooded rats. *n* = 7, 9, 8, 11, respectively for brain injured animals receiving low, medium, high drug dose and saline. *n* = 9, 9, 9, 10, respectively for sham animals receiving low, medium, high drug dose and saline	Drug given at 48-h post TBI and continued for 7 days (intra-peritoneal injection).	Vigabatrin treatment did not have any effect on the lesion volume. Vigabatrin did not have any effect on recovery of sensory function post TBI (assessed up to 60 days post injury).

The table includes the model of TBI induction, drug administration timeline, and experimental groups along with the reported histopathological outcomes and effect on functional recovery.

## Benzodiazepines

Benzodiazepines are a group of drugs that potentiate GABAergic neurotransmission (14, 50). They do so by acting on GABA_A_Rs and thereby mediate sedative effects and anti-epileptic action (50). Benzodiazepines are commonly used as anesthetic/sedative agents in clinical TBI and also in the treatment of anxiety, insomnia, and seizures (14, 18, 50). By binding to the benzodiazepine receptor on the GABA_A_Rs, these drugs potentiate the effect of GABA by inducing a conformational change on the receptor (50). Though known to reduce ICP and metabolic demand (14), benzodiazepines have been documented to be deleterious for neuroprotection and known to impede the extent of functional recovery in experimental TBI (19, 22, 39, 41).

Statler et al. (19) have reported that the application of diazepam post CCI in rats did not have any impact on the contusional size and number of the surviving neurons in the CA1 region of the hippocampus. Also, animals treated with diazepam exhibited poor cognitive functioning in the MWM test. In another study (39), midazolam application at 24-h post injury in rats interfered with functional recovery and did not have any effect on the lesion size both measured at 72-h post injury. Diazepam application following a lesion to the neocortex (22) impaired recovery of sensory asymmetry as long as 22 days post injury and this delay to behavioral recovery was prevented by the application of benzodiazepine antagonist Ro 15-1788 (51). In a similar experiment (41), the application of diazepam following anteromedial cortical lesions resulted in impaired functional recovery, increased atrophy of the striatum, and cell death in the substantia nigra pars reticulata. Further, systematic application of flumazenil (at 24-h post injury), a benzodiazepine antagonist improved cognitive performance in MWM task in CCI-injured immature animals (52). Finally, diazepam did not have any effect on cortical tissue loss and the number of degenerating cells (determined by Fluoro-Jade C staining) at 3 days post TBI (38).

Similar to propofol, benzodiazepines application prior to TBI could produce beneficial effects with respect to neuroprotection. In a study (42) that involved injuring rats by the FPI method, animals that received diazepam 15 min prior to the injury were characterized by reduced mortality rate and improvement in functional recovery. However, rats treated with the drug 15 min post FPI, did not exhibit any significant difference in the mortality rate compared to saline-treated animals. For this reason, the timing of diazepam application could play a vital role in neuroprotection and functional recovery post TBI at least in experimental animals (Table 1).

## Barbiturates

Barbiturates are a class of drugs that potentiate post-synaptic GABAergic currents and are hence regarded as GABA_A_R agonists (53). This results in increased hyperpolarization of neurons due to an enhanced influx of chloride ions. Also, barbiturates result in the inhibition or blocking of AMPA receptors (14). Barbiturates include drugs such as phenobarbital, thiopental, pentobarbital, methohexital, etc. (53). In experimental TBI, the use of barbiturates post injury has been reported to worsen the injury and impede the pace of behavioral and functional recovery (19, 23). For example, the application of pentobarbital post TBI in rats resulted in no change to lesion volume similar to the effect of some of the other commonly used drugs for anesthesia (19). Additionally, the use of phenobarbital, a barbiturate, post anteromedial lesion to the cortex resulted in delayed behavioral recovery of up to 4 weeks compared to saline-treated animals (23). Therefore, the use of barbiturates could result in impaired functional recovery and may fail to confer neuroprotection benefits similar to the effect of other GABA_A_R agonists.

## Isoflurane

In contrast to the above-mentioned anesthetic agents, isoflurane finds a rare usage in clinical TBI while used extensively in animal research experiments (19). Isoflurane is a volatile anesthetic (54) and several reports from preclinical TBI experiments indicate that isoflurane could be neuroprotective (19–21, 43). In an experiment (19) involving the CCI method of brain injury, isoflurane resulted in better cognitive recovery of rats in the MWM task and also caused better survival rates of CA1 hippocampal neurons. Similarly, in another study (21), rats treated with isoflurane exhibited better performance in motor and cognitive tasks and had significantly reduced secondary damage in the hippocampus. The authors postulated that the neuroprotective effect of isoflurane could be mediated as a result of increased cerebral blood flow and reduced excitotoxicity caused by the anesthetic but the drug had little effect on reducing ICP. In another experiment (43) which compares the anesthesia induced neuroprotective effects of isoflurane and fentanyl, animals treated with isoflurane exhibited significant neuroprotection in terms of the surviving CA3 hippocampal neurons and scored better in functional recovery. Luh et al. compared the effect of 15-min anesthesia in CCI-injured rats and reported that animals treated with isoflurane were characterized by reduced contusional volume and better functional recovery as measured by neurological severity score (20). In a study that involved mild TBI induction by CCI, isoflurane anesthesia resulted in reduced axonal injury (44). Although the above studies indicate a beneficial effect of isoflurane, prolonged isoflurane exposure or deep sedation (47) is reported to cause increased edema (water content) formation post TBI (45, 46).

One of the proposed reasons for isoflurane's neuroprotection could be the drug's multifaceted mechanism of action. Isoflurane counters excitotoxicity by inhibiting glutamate release (54), blocking voltage-gated sodium channels (55) and glutamate receptors (56), prevents calcium (56) entry by blocking NMDA receptors, and maintains perfusion by increasing the cerebral blood flow (21, 57) in addition to its known mechanism of augmenting GABAergic currents. Therefore, based on the results from above-mentioned scientific studies (Table 1), isoflurane could act as a better neuroprotective agent compared to other drugs that act on GABA_A_ receptors although its efficacy in humans remains to be determined.

## Other drugs

Two other drugs that augment chloride currents through GABA_A_Rs are topiramate (58) and vigabatrin (49). Though not used as an anesthetic agent in clinical TBI, both these drugs are well-known for their anti-epileptic efficacy and used widely for controlling seizures (49, 58). Topiramate is a relatively new anti-epileptic drug and acts by potentiating GABAergic neurotransmission, blocking voltage-gated sodium channels, and also acts as an antagonist of AMPA receptors (58). Topiramate applied at various time points post TBI was not effective in reducing edema and did not have any effect on histopathological damage and CA3 cell counts (48). Vigabatrin, a drug that potentiates GABAergic neurotransmission by inhibiting GABA-T (GABA-Transaminase) did not have any effect on the recovery of sensory function post TBI in rats (49).

Bolstering the above-mentioned studies, the application of GABA itself to the injured brain tissue delayed recovery to function in an animal model of hemiplegia (59). Additionally, the application of muscimol, a GABA_A_R agonist, to the brain region (sensorimotor cortex) adjacent to lesion in the anteromedial cortex impacted the long-term recovery of behavioral function (60). Also, the application of pentylenetetrazol (61) a GABA_A_R antagonist following unilateral lesions to the sensorimotor cortex of rats promoted recovery of the functional deficits created by the injury. Therefore, drugs that potentiate GABAergic neurotransmission might not exert neuroprotective benefits and may impair functional recovery post TBI in animal studies.

## Discussion

Even though numerous animal studies have recorded the deleterious effect of GABAergic drugs on neuroprotection, it should be noted that these agents are some of the commonly utilized drugs for sedation in clinical TBI (14, 62). Propofol is widely used for sedation in TBI patients owing to its well-established safety profile and effect on reducing ICP (14, 62). Next to propofol, benzodiazepines find their application extensively in clinical TBI (prior to the advent of propofol) owing to their ability to increase the seizure threshold and reduce ICP (14). Although benzodiazepines are associated with delirium, refractoriness, and withdrawal effects, it is no different (midazolam) from propofol with respect to its effect on hemodynamic variables (14, 63). Propofol is known for faster wake-up times and better quality of sedation (14, 63).

Barbiturates, once used for pharmacological sedation in clinical TBI cases are now replaced by other drugs like propofol (64, 65) owing to their adverse side effects. However, the Brain Trauma Foundation (BTF) (66) recommends the use of barbiturates for the management of refractory increased ICP in clinical TBI even though there have been reports of uncontrolled ICP (67, 68) and hypotension (67) by this class of drugs. In a retrospective study of trauma patients (69), the use of barbiturates within 24-h of hospital admission is associated with increased mortality. Hence, these drugs require very cautious application especially in severe cases of clinical TBI.

Isoflurane, though widely employed in preclinical TBI experiments, is rarely used in clinical TBI compared to other anesthetics (70). As mentioned in this review, isoflurane is found to be neuroprotective in a number of animal studies (19–21, 43). One of the reasons for sparse usage in clinical TBI is that isoflurane being a vasodilator may result in elevated ICP through its effect on cerebral blood flow (70, 71). In addition to that, the long-term effects of isoflurane treatment in the clinical TBI population is not well-documented in the literature (70). Concerns range from short-lived effects on cerebral injury (72) to little or no impact on functional recovery (73). Large, multi-center clinical trials on brain injury patients can help answer isoflurane's effect on neurological function over a longer time period. Other reasons could be specific to the use of volatile anesthetics such as issues with air pollution and the need for a specialized ventilating device (70, 71).

The reason for the poor efficacy of the drugs discussed in this study could be linked to changes in GABAergic neurotransmission in the posttraumatic brain. Almost all the drugs discussed in the study exert their action by interfering with neuronal GABAergic currents. The inhibitory action of the neurotransmitter GABA is mediated by chloride ions and is developmentally regulated (74–77). GABA is excitatory in immature neurons but its inhibitory action is restored during the course of development (75, 77). The inhibitory efficacy of GABA which depends on the concentration of intracellular chloride ions is controlled by the opposing action of two cation chloride transporters: NKCC1 and KCC2 (78). The expression levels of both these transporters vary across the development with NKCC1 transporters abundantly present in immature animals but their expression levels are greatly decreased in an adult brain (76). On the other hand, the expression of KCC2 is reduced at birth but increases during the post-natal developmental stages (76, 79).

Post TBI, changes in the expression levels of NKCC1 and KCC2 transporters have been reported in a number of preclinical studies (80–83). According to a study involving TBI in mice, the expression levels of NKCC1 co-transporters were upregulated until 24 hours post injury (80). Similar results depicting the upregulation of NKCC1 co-transporters were reported in a study that employed a closed head injury model (81) and weight drop method (82) to induce TBI in animals. Similarly, downregulation in the expression levels of KCC2 transporters has also been reported in the TBI literature (81, 83).

An immediate outcome of upregulation of NKCC1 and/or downregulation of KCC2 co-transporters post TBI is increased intracellular chloride levels leading to depolarized values of GABA (chloride) reversal potential. Confirming this theoretical observation, depolarized values of chloride reversal potential have been reported in numerous preclinical TBI studies (81, 83, 84). Therefore, GABA might cause paradoxical excitation (instead of inhibition) post TBI which might explain the inefficacy and possible detrimental effects of GABA_A_R agonists post TBI. A number of experimental studies (80–82, 85–88) and a recent computational study (89) have supported this hypothesis for the relative inefficacy of GABA post TBI. In these studies, blocking NKCC1 co-transporters by a drug called Bumetanide, a diuretic, caused neuroprotection and reduced edema formation in the brain. Also, pairing GABA_A_R agonists with Bumetanide might restore the inhibitory efficacy of GABA in post-traumatic brain states (89, 90). In addition to TBI, Bumetanide has been shown to reinstate the inhibitory action of GABA in other pathological brain conditions (91–93).

Another potential mechanism that could explain the adverse effects of GABA and GABAergic drugs post brain trauma is the depolarizing gradients exerted by bicarbonate ions (89, 94, 95). GABA_A_ receptors conduct not only chloride ions but also bicarbonate ions with the latter contributing to about 20% relative permeability (96). Regeneration of bicarbonate gradients is firmly controlled by pH buffers (97). However, when bicarbonate resting gradients were allowed to break down either experimentally (94) or in a computational model (89), the GABA mediated depolarization was reduced significantly suggesting a potential role for bicarbonate signaling in this process.

Changes in the subunit composition of GABA_A_ receptors post TBI could also contribute toward the neuroprotective inefficacy of GABAergic drugs (98, 99). In experimental TBI, changes in the expression pattern of GABA_A_ receptor subunits have been reported at various time points following injury (98, 99). Furthermore, calcium-dependent enhancement of GABAergic currents following trauma (100) in conjunction with depolarizing chloride gradients discussed above could partly explain the deleterious effect of GABAergic drugs post TBI. Lastly, decreased binding capacity of GABA_A_ receptors (101), extensive dendritic damage and spines leading to loss of receptors (102) could potentially reduce the therapeutic efficacy of GABAergic drugs following brain trauma.

In contrast to the reported neuroprotective inefficacy of GABAergic drugs in TBI, dexmedetomidine (Dex), a novel drug that works as an agonist of α2–adrenoreceptor (103) has shown to be neuroprotective in numerous animal studies (40, 104–109). For example, Dex has been shown to reduce neurodegeneration following CCI in mice (104, 107). Similar effects of Dex on brain edema were reported in other preclinical animal studies (40, 105, 106, 108, 109). This could be because of the fact that Dex hyperpolarizes neurons not by targeting GABAergic neurotransmission but through other means (acting on inwardly rectifying potassium channels) (103).

It's worthwhile to bring to the attention of the readers that results from TBI animal experiments may not always produce the same effect in humans (110). Even though preclinical studies have documented the therapeutic efficacy of numerous drugs for neuroprotection, disappointing results have been observed in Phase III clinical trials (110). For example, progesterone was shown to exhibit therapeutic and functional benefits in animal studies (111), but clinical trials of the drug on humans have failed to yield any significant effect (112). Therefore, the application of the results of this review in clinical TBI might have its own limitations given the poor success rate of replication of TBI animal studies on humans. Nevertheless, this study could be used as a motivation factor for more research to gain an increased understanding of the effects of GABAergic drugs in clinical TBI.

## Conclusion

A careful review of preclinical TBI literature has highlighted the inefficacy and possible anti-neuroprotective action of some of the anesthetic agents that augment GABAergic currents and are commonly used in clinical TBI. With the exception of isoflurane, all other anesthetic agents that augment GABAergic neurotransmission might not cause neuroprotection and, in many cases, could be detrimental to it and may impede functional recovery. Changes in the expression patterns of chloride transporters post TBI could be a possible reason behind the unexpected action of such drugs. Until a better understanding emerges about their neuroprotective efficacy in humans, adequate care should be exercised for their application in clinical TBI.

## Author contributions

SKS conceptualized the research topic, performed literature search and analysis, and wrote the manuscript.
